# Positive cross-linguistic influence in the representation and processing of sentence-final particle *le* by L2 and heritage learners of Chinese

**DOI:** 10.3389/fpsyg.2023.1145493

**Published:** 2023-07-25

**Authors:** Shanshan Yan, Ziyin Mai, Yang Zhao

**Affiliations:** ^1^School of Chinese as a Second Language, Peking University, Beijing, China; ^2^Department of Linguistics and Translation, City University of Hong Kong, Hong Kong, China

**Keywords:** positive cross-linguistic influence, bilingual co-activation, SFP *le*, L2 learners, heritage learners

## Abstract

This study investigates the representation and processing of written Chinese sentences subject to a semantic condition (i.e., “direction of change”) attached to the sentence-final particle (SFP) *le* in Mandarin Chinese. Three groups of bilingual speakers of Chinese and English who differ in their onset age of bilingualism and proficiency of English were studied. It was anticipated that there would be a positive cross-linguistic influence (CLI) from English due to similarities between the SFP *le* and the English adverb *already* in terms of direct semantic transfer. An acceptability judgment (AJ) task and a self-paced reading (SPR) task were conducted to elicit judgment and processing difficulty with and without semantic violations. The participants included English-dominant second language (L2) learners (*n* = 18) and heritage learners (*n* = 19) who had advanced proficiency in Chinese, as well as monolingually raised Mandarin speakers from China as a baseline control group (*n* = 18). The results indicated that sensitivity to violations of the semantic condition varied depending on factors such as the specific structure (noun vs. verb phrase), the task type (offline vs. online), and the type of bilingual speaker (early vs. late). Among the three groups of bilinguals, the heritage learners demonstrated a representation of the semantic condition that resembled the target language across different sentence structures, whereas the L2 learners did not. Furthermore, the heritage learners exhibited earlier sensitivity to violations during online processing compared to the baseline control group. These exceptional results can be attributed to the heritage learners’ early exposure to and positive CLI between the SFP *le* in Mandarin and the English adverb *already*.

## 1. Introduction

Cross-linguistic influence (CLI), in which grammatical properties of prior or simultaneously acquired language(s) influence those of subsequently acquired ones, is widely reported (see Lago et al., 2021 for a review). Moreover, first language (L1) properties, if transferred into the second language (L2), may persist into very advanced stages of L2 acquisition (Inagaki, 2001; Rankin, 2014; Bauke, 2020; among others). In sentence reading tasks which necessarily involve real-time information processing, coordination of grammatical properties from various linguistic domains such as syntax and semantics can be cognitively taxing (Hopp, 2006). Most studies on CLI have focused on the negative effects of CLI (Odlin, 2001; Yuan, 2004; Yuan and Zhao, 2005; Jackson and Dussias, 2009; among others). However, much less emphasis has been placed on its positive side, which should facilitate both the acquisition and processing of the target language (Odlin, 1989; Yip et al., 2018). Furthermore, for structures that pose similar constraints cross-linguistically, the syntactic differences between them, for example whether less embedded ones exert larger positive CLI effects, are unknown. For L2 learners (L2ers), online reading tasks are more challenging than offline ones as explicit knowledge is less accessible by the parser in online tasks due to time pressure (e.g., Jiang, 2004; Andersson et al., 2019; Mickan and Lemhöfer, 2020). However, for heritage language learners (HLers), early exposure to the target language might to some extent bring about advantages over adult L2 learners in terms of sensitivity to very subtle semantic features (e.g., Mai and Deng, 2017; Polinsky and Scontras, 2020). In this study, we examine whether and to what extent positive CLI takes place in establishing and maintaining a syntax-semantic condition in the L2 and heritage Mandarin Chinese using both offline and online reading tasks.

## 2. CLI in grammatical acquisition and sentence reading

### 2.1. Structural effects on CLI

When the target structure in the L2 does not have a close structural equivalent in the L1, or the L1 equivalent is substantially different from the L2 target **(“L1-L2 different structures”)**, positive CLI from the L1 on the L2 is not expected. Nevertheless, L1-L2 similarity is by no means a necessary condition for native-like sentence processing in the L2. Frenck-Mestre and Pynte (1997) showed that in processing structurally ambiguous English sentences (i.e., subject/object ambiguities, non-ambiguous in French), adult French-L1 English-L2 bilinguals performed in a similar manner to English monolinguals despite temporary difficulties. In Hoover and Dwivedi (1998), French clitics in causative and non-causative sentences were successfully acquired by highly proficient adult English-L1 learners of French. Similarly, Jackson and Dussias (2009) revealed that L2 learners of German, when reading *wh*-extractions in German, were sensitive to morphological case-marking not instantiated in their L1 English (other studies see Hasegawa et al., 2002; Yokoyama et al., 2006).

Nevertheless, when the L2 target structure has a close equivalent in the L1 **(“L1-L2 similar structures”)**, it is expected that structural similarity should lead to positive L1 transfer, facilitating the acquisition of the L2. However, the expected positive CLI is not always present (Bates and MacWhinney, 1989; Tokowicz and MacWhinney, 2005; Jing, 2008; Sabourin and Stowe, 2008, among others). For example, native speakers of Greek showed clear preferences in terms of high/low attachment in interpreting nouns in relative clauses, which is shared by a number of languages such as Spanish, German, and Russian, and are thus not expected to cause problems for Greek L2 learners whose L1 is any of these three languages. However, Papadopoulou and Clahsen (2003) found that L2 learners of Greek who were highly proficient in it, showed no preference for high/low attachment in the structure, suggesting the L1-L2 similarity might not play any facilitating role. Similar findings were reported with regard to the comprehension of non-local dependencies (Felser and Roberts, 2007; Omaki and Schulz, 2011).

Jeong et al. (2007) and Jeong et al. (2007) studied the processing of word order and related syntactic features (e.g., case-marking) by native speakers and L2 learners of SVO languages (Mandarin Chinese, English) and SOV languages (Korean, Japanese). Those learning an L2 with a word order similar to their L1 showed advantages in brain activities in processing the L2 compared with those learning an L2 with a word order different from their L1, but the advantage seems to be specific to the task adopted (i.e., a behavioral auditory sentence comprehension task), indicating task effects on the extent of positive CLI, which we will elaborate on in the next section.

### 2.2. Task effects on CLI

CLI, whether negative or positive, is extensively investigated in both **online** processing and **offline** comprehension and judgment tasks, and the findings are mixed. Jiang (2004) investigated subject-verb agreement marking in English by Chinese-L1 learners of L2 English. The L2 learners were quite accurate in an offline written test, yet insensitive to the same morphosyntactic manipulation in an online comprehension task. Elsewhere, Hopp (2006) showed that lower-proficient advanced German L2 speakers (whose L1s were English and Dutch) were able to utilize case-marking information as evidenced by comprehension accuracies, but were insensitive to the same information in online processing. However, in Turkish-L1 and German-L1 learners of L2 Dutch, CLI in pronoun resolution is observed in offline interpretation tasks yet absent in online reading tasks (Roberts et al., 2008). In Hopp and Grüter (2023), L1 effects were identified among German-L1 and Japanese-L1 L2 learners of English in offline comprehension of *wh*-questions rather than in online processing of the same questions (for more evidence on the online vs. offline asymmetry, see Kaan et al., 2015; Andersson et al., 2019; Mickan and Lemhöfer, 2020).

In bilingual processing, abundant evidence has supported **co-activation** of both languages in various types of bilinguals, even when the linguistic stimuli are presented monolingually (e.g., De Groot, 2011; Hopp and Grüter, 2023). Most studies have examined cross-linguistic co-activation at the **lexical** level, with a special focus on L1-L2 equivalents that are cognate words (Costa et al., 2000; Dijkstra, 2005). Processing these words in the L2 activates their L1 equivalents and facilitates lexical processing in the L2 (Dekydtspotter et al., 2006; Miller, 2014). However, less is known about the activation of **syntactic** structures in bilingual processing, except Hopp (2017) and Vaughan-Evans et al. (2020). Hopp (2017) touched upon syntactic co-activation in German-L1 learners of English through lexical co-activation in reading. Specifically, he investigated whether English relative clauses with different word orders that overlap with German-L1-embedded clauses activates the L1 grammars in online L2 sentence comprehension. His findings suggest the L1 word order was co-activated. Vaughan-Evans et al. (2020) examined Welsh-L1 English-L2 early bilinguals’ online reading of English sentences with manipulated morphosyntactic rules (i.e., the Welsh soft mutation rule) and proposed that co-activation of the L1 syntax may occur through syntactic rules. As for the processing of complex syntactic features, such as syntax-semantic relationships investigated in the current study, much work is still needed.

### 2.3. Learner effects on CLI

It has been shown that bilingual co-activation may boost the processing of relevant syntactic units. However, this positive effect is not found across types of bilinguals. For instance, Canseco-Gonzalez et al. (2010) compared three groups of Spanish-English bilinguals with various onset ages of bilingualism and proficiency in Spanish: the early bilinguals (with an onset age of both languages before 6 years) and the English-L2 group (onset age after 6 years) co-activated Spanish in word recognition tasks in English, whereas the Spanish-L2 group (onset age after 6 years) did not reveal any co-activation effect. The authors argued that the more proficient the early bilinguals are, the more successful and accustomed the learners are in terms of co-activation. This leads to an interesting prediction: early bilinguals of Languages A and B may be more efficient than late bilinguals (e.g., L2 learners of Language A) in co-activating Language B in reading in Language A. Moreover, early bilinguals may even outperform monolingually raised speakers of Language A, who are late bilinguals of the same languages. Our study follows up on this line of research and investigates learner effects in three types of proficient bilinguals in Chinese and English.

**Heritage language speakers (HLers)** are typically early bilinguals who acquire the HL as their L1 (or one of their L1s alongside the societal majority language) through interaction with parents who are native speakers of the language in naturalistic settings (Polinsky and Scontras, 2020). Because an HL is by definition a minority language in the larger community, adult HL speakers usually (but not always) develop stronger abilities in the societal majority language through mainstream schooling and possess varying proficiency in the HL (Montrul, 2016). Some HL speakers are able to develop literacy in the HL through bilingual education programs, community schools, or language courses. **L2 learners (L2ers)** are typically late bilinguals who acquire their L1 at early childhood and are raised in the L1 context. Their L2 was mostly learned through formal instruction after puberty. HLers, on the one hand, can display substantial differences from L2ers in many acquisition and processing aspects across linguistic domains (Montrul, 2016); on the other hand, they are also different from monolingually raised speakers of that language (“baseline”) in judging and processing complex grammatical properties of the HL (e.g., Mai and Deng, 2017; Polinsky and Scontras, 2020). For HLers, it is likely that the experience of developing both the HL and the majority language concurrently in childhood gives rise to more accurate and accessible mapping between corresponding structures in the two languages and bring advantages over L2 and monolingual baselines in L1-L2 similar structures. In this sense, it might be possible that the CLI is modulated by learner effect, and we further explore this in the current study. In comparing English-dominant L2 and HL Spanish speakers, Regulez and Montrul (2023) found great learner variations regarding acceptability, production, and online comprehension in Spanish differential object marking, reporting the presence of task effects. This study tests L1-L2 similar structures involving complex syntax-semantics coordination, the sentence-final particle (SFP) *le* in Mandarin Chinese and the adverb *already* in English through both offline and online tasks.

## 3. The SFP *le* in Mandarin Chinese and the adverb *already* in English

### 3.1. The SFP *le*

Mandarin Chinese has a rich set of SFPs, among which *le* denotes a newly obtained state or “change of state” (Chao, 1968; Li and Thompson, 1981; Paul, 2015; Paul and Yan, 2022) and entails that the current state does not hold before speech time (Soh, 2009).^1^ When the change is associated with a naturally occurring or commonly perceived ordered **scale** (e.g., numbers in ascending order, or stages of human development), the SFP *le* imposes, among other conditions, a semantic constraint on the direction of change encoded, as outlined in (1):

1. The change must take place from an earlier/lower point to a later/higher point along the associated scale, rather than vice versa (Xiang, 1998; Xing, 2001).

For example, in stative [*be*-NP] structures as presented in (2) and (3), when the NP identifies a point which is straightforwardly interpretable as a high point on the ordered scale, entailing at least one lower point on the scale (termed as **“higher NP,”** e.g., where *dà-háizi* (‘big child’) is a point higher than *xiǎo-háizi* (‘little child’) on the scale of human development), [*be*-NP] is naturally compatible with the SFP *le*, as shown in (4). However, when the NP denotes a low point on the scale without the entailment of an even lower point (termed as **“lower NP,”** e.g., *xiǎo-háizi* (‘little child’)), [*be*-NP] is less compatible with the SFP *le*, as in (5). Notice that both types of NP are natural without the SFP *le*, as illustrated in (2) and (3).

(2) Higher NP in [*be*-NP]
  Xiǎowáng shìdàháizi.
  Xiaowang COPbigchild
  ‘Xiaowang is a big child.’
(3) Lower NP in [*be*-NP]
  Xiǎowáng shìxiǎoháizi.
  Xiaowang COPsmallchild
  ‘Xiaowang is a small child.’
(4) Higher NP in [*be*-NP-*le*_SFP_]
  Xiǎowáng shì **dà háizi le**.
  Xiaowang be big child LE
  ‘Xiaowang is a big child already’.
(5) */?Lower NP in [*be*-NP-*le*_SFP_]
  */?Xiǎowáng shì **xiǎo háizi le**.
  Xiaowang be small child LE
  */?‘Xiaowang is a small child already.’

Another manifestation of (1) can be found regarding the SFP *le* with **upward/downward-entailing duration phrases in [V-*le***_**ASP**_**-duration-*le***_**SFP**_**] structures**. Upward-entailing contexts identify, on a scale, the lowest possible value that is true for the relevant proposition and entail values higher than this point [e.g., *zhìshǎo wǔnián* (‘at least 5 years’), or *chāoguò sāntiān* (‘exceeding 3 days’)]; they are natural with the SFP *le*, for the direction of the entailment aligns with the direction of change as stipulated by the SFP *le* above, as illustrated in (8). In sharp contrast, **downward-entailing contexts** identify the highest point that holds for the relevant proposition [e.g., *búdào wǔnián* (‘less than 5 years’), or *zuìduō sāntiān* (‘at most 3 days’)] and are thus not compatible with the SFP *le*, as depicted in (9). Notice here that both upward and downward elements are possible in sentences without the SFP *le*, as demonstrated in (6) and (7).^2^

(6) Upward-entailing duration in [V-*le*_ASP_-duration]
  Wǒ zài zhèr[_VP_ zhù le**zhìshǎo** wǔ nián].
  1SG at herelive PERF at-least five CL
  ‘I lived here for at least 5 years.’
(7) Downward-entailing duration in [V-*le*_ASP_-duration]
  Wǒ zài zhèr[_VP_ zhù le**búdào**wǔ nián].
  1SG at herelive PERF less-than five CL
  ‘I lived here for less than 5 years.’
(8) Upward-entailing duration in [V-*le*_ASP_-duration-*le*_SFP_]
  Wǒ zài zhèr[_VP_ zhù le**zhìshǎo** wǔ nián] **le**.
  1SG at herelive PERF at-least five CL LE
  ‘I have lived here for at least 5 years already (which was not the case before).’
(9) */?Downward-entailing in [V-*le*_ASP_-duration-*le*_SFP_]
  */?Wǒ zài zhèr[_VP_ zhù le**búdào**wǔ nián] **le**.
  1SG at herelive PERF less-than five CL LE
  */?‘I have lived here for less than 5 years already.’
  [slightly modified examples from Soh (2009, p. 638–641)].

### 3.2. The adverb *Already* in English

Lacking SFPs, English has an adverb *already*, which is also subject to a “change of state” interpretation and is considered a semantic counterpart of the SFP *le* in Mandarin in many contexts (Traugott and Waterhouse, 1969; Soh, 2009). It has been argued that the English adverb *already* entails a “cognitive modeling of event sequence” including canonical courses of development (Michaelis, 1992, 1996). As shown in the translations in (4), (5), (8), and (9), the acceptability of *already* in English is subject to the same restrictions as the SFP *le* in contexts with scalar inferences: upward-entailing contexts (e.g., *at least*) and higher NPs (e.g., *big child*) are clearly more acceptable than downward-entailing contexts and lower NPs (e.g., *less than,* or *small child*).

In the absence of literature on cross-linguistic mapping between the SFP *le* and the English adverb *already* in Mandarin-English bilinguals, we searched the literature for evidence in Chinese-English bilinguals in general. Szeto et al. (2017) found that Cantonese-English bilinguals associated the English adverb *already* with the Cantonese SFP *laa3* and aspect marker *zo2*, which are well-established counterparts of the SFP *le* and aspect marker *le* in Mandarin.

## 4. This study

### 4.1. Research questions and predictions

As reviewed above, L1-L2 similarity facilitates both acquisition and processing due to positive CLI and bilingual co-activation, but whether positive CLI happens in different types of bilingual speakers and in complex grammatical processing involving semantic computation is an empirical question that has been raised only recently in studies. To fill this gap, this study looked into the acquisition and processing of higher/lower NPs in [*be*-NP-*le*_SFP_] and upward/downward-entailing duration phrases in the [V-*le*_ASP_-duration-*le*_SFP_]. The following two research questions guided this study:

1. Given cross-linguistic similarities between the SFP *le* in Mandarin and the adverb *already* in English, will English-L1 learners of L2 Mandarin **(L2ers)** succeed in differentiating, in the SFP *le* sentences, the acceptability between higher and lower NPs such as in (4) and (5) and between upward- and downward-entailing duration phrases such as in (8) and (9) in both offline judgment and online reading?
2. Will adult **HLers**, who have acquired both Chinese and English at a young age, outperform proficiency-matched L2ers and pattern with monolingually raised Chinese L1 speakers in China (**baseline controls**), due to the early onset of Chinese-English bilingualism?

For English-L1 L2ers, consistent differentiation between the more acceptable and the less acceptable sentences hinges upon the mental representation of the semantic condition regulating the use of the SFP *le* in (1), which may be formed based on absorbing patterns in the input and profiting from positive transfer from *already*, if the mapping between the SFP *le* and *already* is established. We predict that L2ers will be able to make the differentiation, but they may be more successful in recognizing violations caused by lower NPs in [*be*-NP-*le*_SFP_] than downward-entailing duration phrases in [V-*le*_ASP_-duration-*le*_SFP_], because the NPs in the former structure are less embedded, linearly closer to the SFP *le*, and semantically more concrete than the duration phrases in the latter. For HLers, we predict that they will outperform their L2 peers due to the early onset of bilingualism and likely early mapping between the SFP *le* in Mandarin and the adverb *already* in English and subsequently stronger facilitative effects. To test these predictions, we conducted an experiment consisting of an offline acceptability judgment task and an online self-paced reading task.

### 4.2. Participants

The data of this study are from a larger project investigating the acquisition and processing of several grammatical structures by adult L2 and HL learners of Mandarin. The participants were recruited from Beijing, Shanghai, and Hangzhou in China, and Cambridge in the United Kingdom. A language background questionnaire was administered to collect information about each participant’s language experience. The original sample consists of data from 150 participants. Given the research goals of this study and the complexity of the grammatical construction under investigation, we selected from the original sample participants who self-reported having intermediate or above-intermediate proficiency in written Chinese. Other criteria included: to qualify as L2ers, the participant must have reported English as their native language, and started to learn Chinese after puberty (average onset age 18 years, see Table 1); and to qualify as HLers, the participants must have reported early and substantial exposure to both Chinese and English in an English-speaking country and self-rated English as their dominant language at time of data collection. Ultimately, 18 L2ers (6 females) and 19 HLers (13 females) satisfied the above criteria and were included in this study.

**Table 1:** Participant information (SD in brackets).

Groups	*N*	Age	Onset age	Months studying Chinese	Months in China	Cloze test
L2	18	23 (2.2)	18 (4.6)	60 (37.8)	38.1 (47.4)	31.3 (1.8)
HL	19	22 (2.9)	0	187 (102.3)	23.2 (28.9)	33.2 (2.4)
Baseline	18	23 (4.5)	0	N/A	N/A	38.8 (1.1)

The HLers had been exposed to Mandarin Chinese (*n* = 15) or Cantonese and Min Chinese^3^ (*n* = 4) through everyday interactions with family members from birth, with at least one parent being a native speaker of Chinese. They were either born and raised in an English-dominant country (UK, US, etc., *n* = 14) or had immigrated with their family to an English-dominant country before the age of 5 (see Supplementary material for each HLer’s language exposure). The baseline controls (*n* = 18) were undergraduate or graduate students at universities in Beijing (12 females), majoring in liberal arts (*n* = 10) or science (*n* = 8). They were all born and raised in northern China; none had visited an English-speaking country by the time of data collection.

An established cloze test (Zhao, 2006; Yuan, 2010; Mai and Yuan, 2016; etc.) was adopted to gauge the learners’ reading proficiency in Chinese. The test included two short passages with 40 blanks and participants were required to fill in each blank with a Chinese character. No Pinyin (romanized form of Chinese) was provided. ANOVA and *post hoc* Tukey tests indicated that both the L2ers and HLers were significantly different from the baseline controls [*F*(2, 52) = 78.24, all *p* < 0.000], but they were not significantly different from each other (*p* = 0.921), suggesting comparable proficiency between the L2ers and the HLers. Information on the participants (*n* = 55) is summarized in Table 1.

### 4.3. Tasks

#### 4.3.1. Acceptability judgment (AJ) task

In the AJ task, the participants judged the acceptability of four types of sentences on a four-point Likert scale (ranging from ‘completely unacceptable,’ to ‘probably unacceptable,’ to ‘probably acceptable,’ and to ‘completely acceptable’):

Higher and lower NPs in sentences with and without the SFP *le*:

Type 1 (*n* = 4): [*be*-NP_HIGHER_-*le*_SFP_], as in (4),

Type 2 (*n* = 4): */?[*be*-NP_LOWER_-*le*_SFP_], as in (5),

Type 3 (*n* = 4): [*be*-NP_HIGHER_], as in (2), and

Type 4 (*n* = 4): [*be*-NP_LOWER_], as in (3).

Upward/downward-entailing duration phrases with and without the SFP *le*:

Type 5 (*n* = 4): [V-*le*_ASP_-Duration_UP_-*le*_SFP_], as in (8),

Type 6 (*n* = 4): */?[V-*le*_ASP_-Duration_DOWN_-*le*_SFP_], as in (9),

Type 7 (*n* = 4): [V-*le*_ASP_-Duration_UP_], as (6), and

Type 8 (*n* = 4): [V-*le*_ASP_-Duration_DOWN_], as in (7).

Four tokens were constructed for each type, rendering a total of 32 tokens. These critical items were mixed with 128 other items testing other grammatical structures such as *wh*-questions in the larger study. The sentences were randomized and presented one-by-one on a computer screen. The participants pressed one of five designated keys on the keyboard to indicate their rating on the scale (the four-point scale plus a separate “I do not know” option), and the program was designed such that once the testing has started, the participant could see only one sentence on the screen and they could not return to a pervious item or change their answers. Six additional training items were provided before the critical items.

#### 4.3.2. Self-paced reading (SPR) task

In this “read and judge” task, the participants read a critical sentence with the SFP *le* and provided a binary truth-value judgment (“correct” or “incorrect”) on a follow-up comprehension sentence based on their understanding of the previous SFP *le*-sentence. Unlike previous studies in which participants were required to read the sentences as quickly as possible, in this task we asked them to read the sentences at a natural and comfortable pace. This is because participants in pilot studies reported considerable anxiety and fatigue when they had to read the sentences as quickly as possible. The test items were presented segment by segment on a computer monitor. Thereafter, participants had to press a key to see the next segment of the SFP *le*-sentence. Upon reaching the end of the SFP *le*-sentence, the comprehension sentence was presented in full for the participants’ judgment.

Four conditions, as shown in (10), (11), (12), and (13), were created to test the two structures, with six or eight trails in each condition. The SFP *le*-sentences in the SPR task were longer than those in the AJ task, with an additional clause following the SFP *le*-clause to allow for spill-over effects and potentially delayed processing of the critical element (the SFP *le*). The sentences were segmented into seven windows for [*be*-NP-*le*_SFP_] and nine for [V-*le*_ASP_-duration-*le*_SFP_] so that only one word (mostly disyllabic, bimorphemic with only a few systematic exceptional cases) was presented to the reader in a window. Note that in [*be*-NP-*le*_SFP_] conditions, the monosyllabic SFP *le* [the critical window, W4 in (10) and (11)] was presented separately from the other words; in [V-*le*_ASP_-duration-*le*_SFP_] conditions, the upward/downward quantifiers were presented in a single time window without the temporal nouns, which were presented together with the SFP *le* in the subsequent window [the critical window, W5 in (12) and (13)]. Half of the comprehension sentences were directly related to the SFP *le*-clause and the other half to the additional clause. In addition, half of them were expected to elicit “correct” responses and the other half “incorrect” ones.

(10) [*be*-NP_HIGHER_-*le*_SFP_] (*n* = 6)

**Table:** 

		pre-critical	critical	1st post-critical	2nd post-critical	
W1	W2	W3	W4	W5	W6	W7
Xiǎomíng	/shì	/dànánhái	/le	/,yīnggāi	/hǎohǎo	/xuéxí.
Xiaoming	COP	big boy	LE	should	well	study
‘Xiaoming is a big boy already; he should study hard.’						
Comprehension sentence: Xiǎomíng bùyīnggāi hǎohǎo xuéxí (‘Xiaoming should not study hard’) Expected response: Incorrect

(11) */?[*be*-NP_LOWER_-*le*_SFP_] (*n* = 6)

**Table:** 

		pre-critical	critical	1^st^ post-critical	2^nd^ post-critical	
W1	W2	W3	W4	W5	W6	W7
Xiǎomíng	/shì	/xiǎonánhái	/le	/,yīnggāi	/hǎohǎo	/xuéxí.
Xiaoming	COP	small boy	LE	should	well	study
Intended: ‘Xiaoming is a small boy, and he should study hard.’						
Comprehension sentence: Xiǎomíng búshì xiǎonánhái (‘Xiaoming is not a small boy’) Expected response: Incorrect						

The 28 SPR items were randomized and mixed with 44 items testing other structures such as aspect markers, negation markers in the larger study using the Latin square design (Dean and Voss, 1999). Two lists (Lists A and B) were generated manually to ensure that the SPF *le*-sentences that were minimally different were not included in the same list. Half of the participants completed List A and the other half List B. In both cases, the test items were divided into two blocks evenly, with an obligatory break between them to reduce the impact of fatigue. Six training items were provided to ensure that the participants understood the instructions.

### 4.4. Procedures

The participants completed both tasks individually in a quiet classroom in one meeting with the first author. Test materials were presented using E-Prime 2.0. Meanwhile, test instructions were given to L2ers and HLers in English, and to Chinese baseline controls in Chinese. An “I do not know” option was available across items throughout the test. The SPR task was administered before the AJ task so that the latter, which activates explicit and metalinguistic knowledge to a larger extent than SPR, did not impact on the SPR results.

## 5. Results

### 5.1. Data coding, trimming, and statistical tools

Judgments in the AJ task were transformed into numerical scores of 1, 2, 3, and 4 for “completely unacceptable,” “probably unacceptable,” “probably acceptable,” and “completely acceptable” respectively. Reading times and responses to comprehension sentences in the SPR task were trimmed and transformed following several steps: (i) responses in the comprehension sentences were checked to screen out participants who did not reach 75% accuracy (following Jiang, 2004; Keating and Jegerski, 2015) – no participant was eliminated in this procedure; (ii) reading times were screened to identify outlying values falling outside an absolute cutoff (shorter than 100 ms or longer than 2000 ms for baseline controls, shorter than 200 ms or higher than 4,000 ms for learners) or a variable cutoff (i.e., three standard deviations above mean reading times of the relevant group), which were then replaced with the absolute cutoff value of the relevant group in statistical analysis (following Keating and Jegerski, 2015); and (iii) to reduce confounding inter-participant differences due to general reading strategies, raw reading times (after adjusting for outlying values as described above) were transformed into **residual reading times**, which reflect differences between the actual raw reading times and the predicted reading times based on the regression equation, with word length as the predictor and raw reading times as the response variable (following Keating and Jegerski, 2015; also known as “deviations from regressions”). After the transformation, positive values indicate longer reading times than predicted by the regression, whereas negative ones mean they are shorter than predicted.

**Linear mixed-effects models (LMMs)** were performed for both tasks in R (version 4.1.1, R Core Team, 2014; *lme4*, Bates et al., 2013). The ratings in the AJ task and reaction times in the SPR task were dependent variables in the respective models. Fixed factors included condition (acceptable vs. less acceptable), group (baseline, L2, and HL), and interactions between condition and group. Random factors included intercepts for subjects and items, as well as by-subject and by-item random slopes. Factors were removed if they did not significantly improve the model according to the maximum likelihood ratio tests. Due to convergence issues, the random slopes for items were removed. Values *t* > |2| were considered statistically significant (following Gelman and Hill, 2007). The results are reported in the following sub-sections.

(12) [V-*le*_ASP_-Duration_UP_-*le*_SFP_] (*n* = 8)

**Table:** 

			pre-critical	critical	1st post-critical	2nd post-critical		
W1	W2	W3	W4	W5	W6	W7	W8	W9
Xiǎowáng	/zài zhèr	/zhù le	/zhìshǎo	/wǔnián le	/, tā	/fēicháng	/xǐhuān	/ zhèr.
Xiaowang	PREP here	live PERF	at least	5 years LE	3SG	very	like	here
‘Xiaowang has lived here for at least 5 years, he likes it here very much.’								
Comprehension sentence: Xiǎowáng xǐhuān zhèr (‘Xiaowang likes it here’) Expected response: Correct

(13) */?[V-*le*_ASP_-Duration_DOWN_-*le*_SFP_] (*n* = 8)

**Table:** 

			pre-critical	critical	1st post-critical	2nd post-critical		
W1	W2	W3	W4	W5	W6	W7	W8	W9
*Xiǎowáng	/zài zhèr	/zhù le	/búdào	/wǔnián le	/, tā	/fēicháng	/xǐhuān	/ zhèr.
Xiaowang	PREP here	live PERF	less than	5 years LE	3SG	very	like	here
‘Xiaowang has lived here for less than 5 years, he likes it here very much.’								
Comprehension sentence: Xiǎowáng méi zài zhèr zhù (‘Xiaowang does not live here’) Expected response: Incorrect								

### 5.2. AJ task

#### 5.2.1. Higher/lower NP conditions

**Figure 1:**
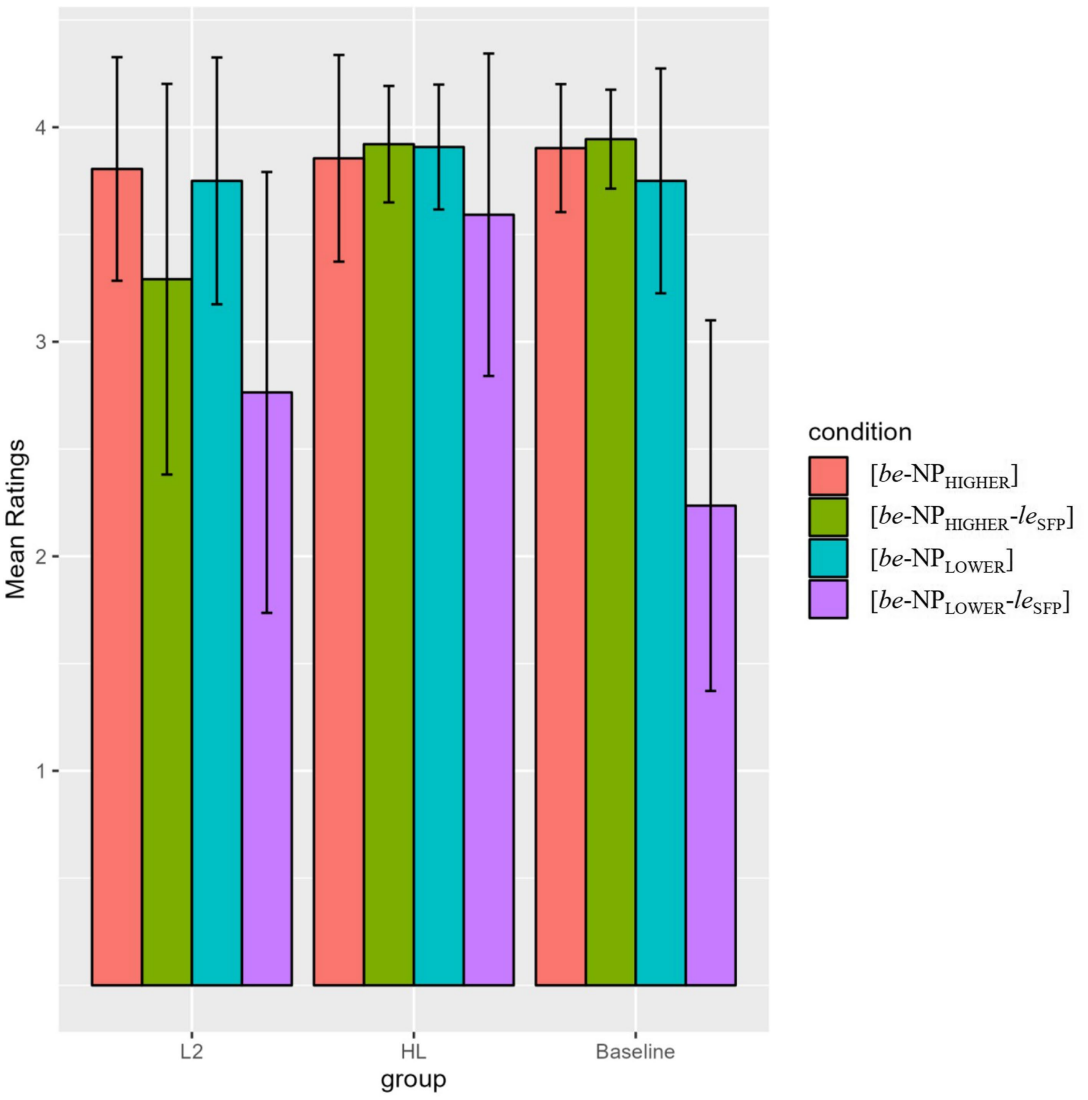
Mean ratings of the higher/lower NP conditions in the acceptability judgment (AJ) task.

As shown in Figure 1; Table 2, all groups rated the [*be*-NP_HIGHER_] and [*be*-NP_LOWER_] sentences at ceiling (means above 3.5), indicating the [*be*-NP] structure is well represented in their Chinese grammars. All groups also rated the [*be*-NP_HIGHER_-*le*_SFP_] sentences higher than minimally different */?[*be*-NP_LOWER_-*le*_SFP_] sentences (*p* < 0.001 for baseline, *post hoc* power = 100%; and *p* < 0.05 for both HL and L2 groups, *post hoc* power for HL = 95.91%, for L2 = 100%).

**Table 2:** Mixed-effects models on [*be*-NP_HIGHER_-*le*_SFP_] and */?[be-NP_LOWER_-*le*_SFP_] in the acceptability judgment (AJ) task.

	Fixed effects		
**Predictors**	**Estimates**	***SE***	***t***
	**L2 learners**		
(Intercept)	2.76	0.12	22.91***
Condition [*be*-higher NP-*le*_SFP_]	0.53	0.13	4.22***
Group Baseline	−0.53	0.16	−3.31**
Group HL	0.83	0.16	5.27***
Condition [*be*-higher NP-*le*_SFP_]: Baseline	1.18	0.15	7.66***
Condition [*be*-higher NP-*le*_SFP_]: HL	−0.20	0.15	−1.31
	**HL learners**		
(Intercept)	3.59	0.12	30.48***
Condition [*be*-higher NP-*le*_SFP_]	0.33	0.12	2.69*
Group Baseline	−1.36	0.16	−8.63***
Group L2	−0.82	0.16	−5.27***
Condition [*be*-higher NP-*le*_SFP_]: Baseline	1.38	0.15	9.07***
Condition [*be*-higher NP-*le*_SFP_]: L2	0.20	0.15	1.31
	**Baseline**		
(Intercept)	2.24	0.12	18.53***
Condition [*be*-higher NP-*le*_SFP_]	1.71	0.13	13.66***
Group HL	1.36	0.16	8.63***
Group L2	0.53	0.16	3.31**
Condition [*be*-higher NP-*le*_SFP_]: HL	−1.38	0.15	−9.07***
Condition [*be*-higher NP-*le*_SFP_]: L2	−1.18	0.15	−7.66***

Reference level for condition = */?[*be*-lower NP-*le*_SFP_], Model formula for baseline group: ratings ~ condition + group + condition:group + (1 | subject) + (1 | item), Model formula for HL group: ratings ~ condition + group + condition:group + (1 | subject) + (1 | item), Model formula for L2 group: ratings ~ condition + group + condition:group + (1 | subject) + (1 | item); ***p < 0.001, **p < 0.01, *p < 0.05.

#### 5.2.2. Upward/downward-entailing duration conditions

**Figure 2:**
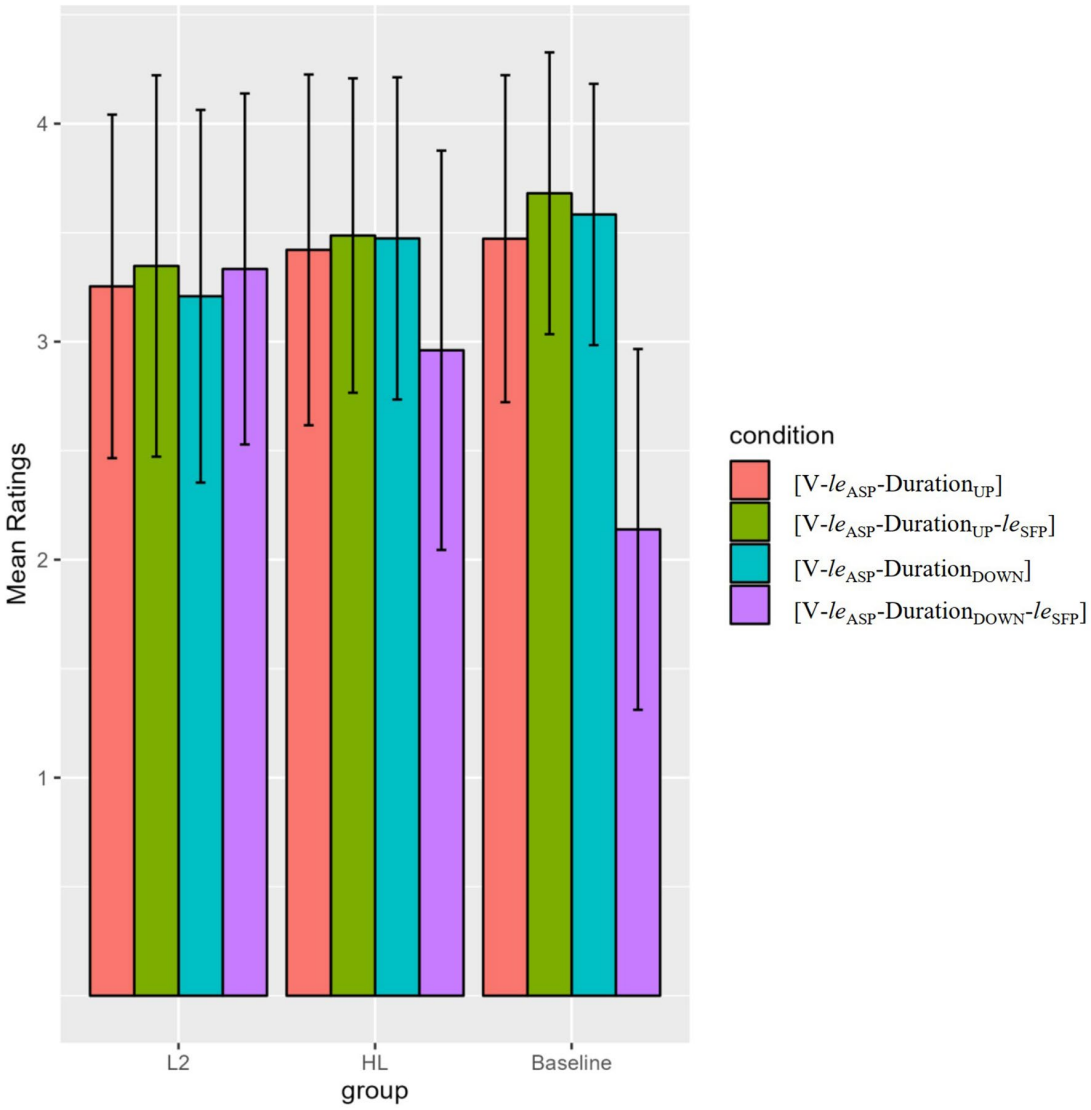
Mean ratings of the upward/downward-entailing duration phrase conditions in the acceptability judgment (AJ) task.

As shown in Figure 2; Table 3, the baseline and HL groups were able to rate [V-*le*_ASP_-Duration_UP_-*le*_SFP_] sentences higher than */?[V-*le*_ASP_-Duration_DOWN_-*le*_SFP_] sentences (*p* < 0.001 for baseline, *post hoc* power = 100%; *p* < 0.01 for HLers, *post hoc* power = 96.14%). However, the L2 group showed no such sensitivity. In addition, all groups rated the [V-*le*_ASP_-Duration_UP_] and [V-*le*_ASP_-Duration_DOWN_] sentences high (means above 3), suggesting their acceptance of the basic upward/downward-entailing duration structures.

**Table 3:** Mixed-effects models on [V-*le*_ASP_-Duration_UP_-*le*_SFP_] and */?[V-*le*_ASP_-Duration_DOWN_-*le*_SFP_] in the acceptability judgment (AJ) task.

		**Fixed effects**	
**Predictors**	**Estimates**	***SE***	***t***
	**HL learners**		
(Intercept)	2.96	0.16	18.03***
Condition [V-*le*_ASP_-Duration_UP_-*le*_SFP_]	0.53	0.20	2.57*
Group Baseline	−0.82	0.20	−4.18***
Group L2	0.37	0.20	1.90#
Condition [V-*le*_ASP_-Duration_UP_-*le*_SFP_]: Baseline	1.02	0.23	4.23***
Condition [V-*le*_ASP_-Duration_UP_-*le*_SFP_]: L2	−0.51	0.23	−2.23*
	**Baseline**		
(Intercept)	2.14	0.17	12.78***
Condition [V-*le*_ASP_-Duration_UP_-*le*_SFP_]	1.54	0.21	7.40***
Group HL	0.82	0.20	4.18***
Group L2	1.19	0.20	6.00***
Condition [V-*le*_ASP_-Duration_UP_-*le*_SFP_]: HL	−1.02	0.23	−4.42***
Condition [V-*le*_ASP_-Duration_UP_-*le*_SFP_]: L2	−1.53	0.23	−6.57***

Reference level for condition = */?[V-*le*_ASP_-Duration_DOWN_-*le*_SFP_], Model formula for baseline group: ratings ~ condition + group + condition:group + (1 + condition | subject) + (1 | item), Model formula for HL group: ratings ~ condition + group + condition:group + (1 + condition | subject) + (1 | item); ***p < 0.001, *p < 0.05, #*p* < 0.1.

To sum up, the AJ task results indicate that like the baseline controls, both HL and L2 learner groups were sensitive to the incompatibility between lower NPs and the SFP *le*. However, only the HLers were able to show sensitivity to the incompatibility between downward-entailing duration phrases and the SFP *le*, patterning with the baseline controls.

### 5.3. SPR task

#### 5.3.1. Higher/lower NP conditions

**Figure 3:**
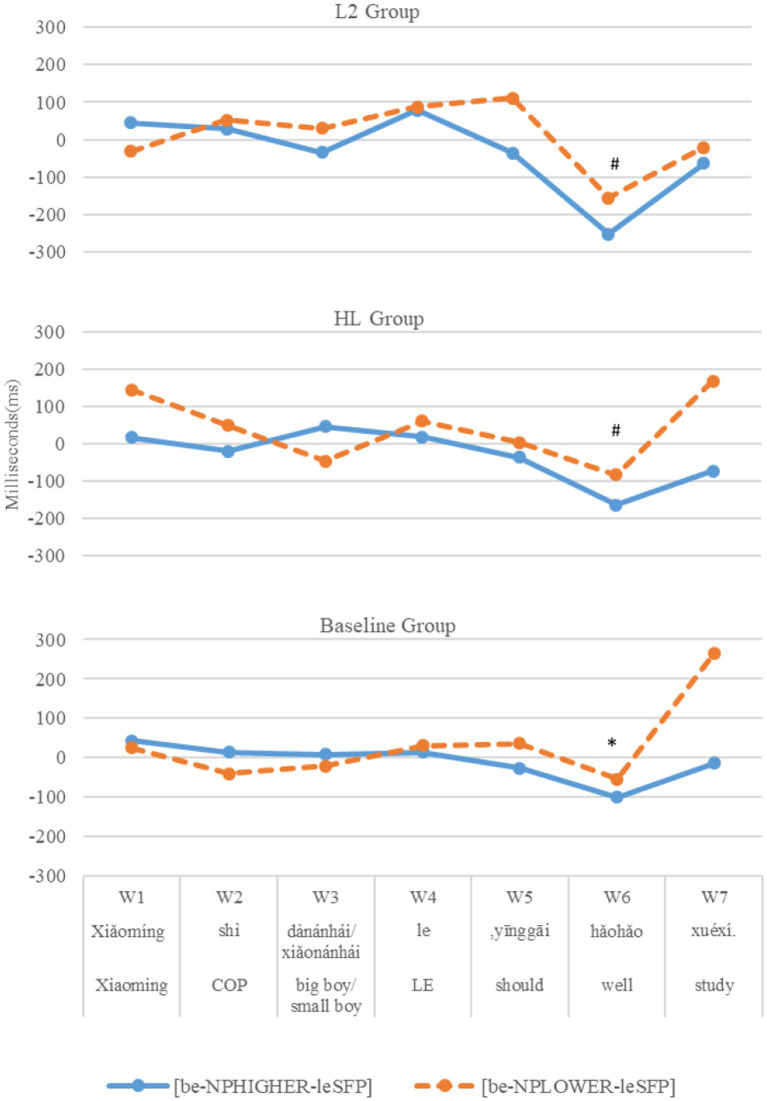
L2, HL, and baseline groups’ residual reading times at each window between [*be*-NP_HIGHER_-*le*_SFP_] and */?[*be*-NP_LOWER_-*le*_SFP_].

Figure 3 presents the reading times at each time window in each condition by each group in three panels. LMM analyses reveal that the three groups of participants showed a similar reading time pattern: no significant differences were found between the two conditions across groups from the pre-critical window (W3) to the first post-critical window (W5); and between-condition differences first showed up in the **second post-critical window** (W6, baseline controls: *p* < 0.05, *post hoc* power = 68.52%; HLers: *p* < 0.1, *post hoc* power = 52.63%; L2: *p* < 0.1, *post hoc* power = 42.59%). Table 4 presents the LMM results of the second post-critical window.

**Table 4:** Mixed-effects models (dependent variable: residual reading time at the 2nd post-critical window [W6]).

	**Fixed effects**		
**Predictors**	**Estimates**	***SE***	***t***
	**L2 learners**		
(Intercept)	−105.21	73.94	−1.42
Condition [*be*-NP_HIGHER_-*le*_SFP_]	−82.69	46.16	−1.79#
	**HL learners**		
(Intercept)	−82.14	29.75	−2.76**
Condition [*be*-NP_HIGHER_-*le*_SFP_]	−80.68	42.07	−1.92#
	**Baseline**		
(Intercept)	−53.81	19.48	−2.76*
Condition [*be*-NP_HIGHER_-*le*_SFP_]	−46.50	18.65	−2.49*

Reference level for condition = */?[*be*-NP_LOWER_-*le*_SFP_], Model formula for baseline group: RT ~ condition + (1 | subject), Model formula for HL group: RT ~ condition, Model formula for L2 group: RT ~ condition + (1 | subject) + (1 | item); **p < 0.01, *p < 0.05, #*p* < 0.1.

#### 5.3.2. Upward/downward-entailing duration conditions

**Figure 4:**
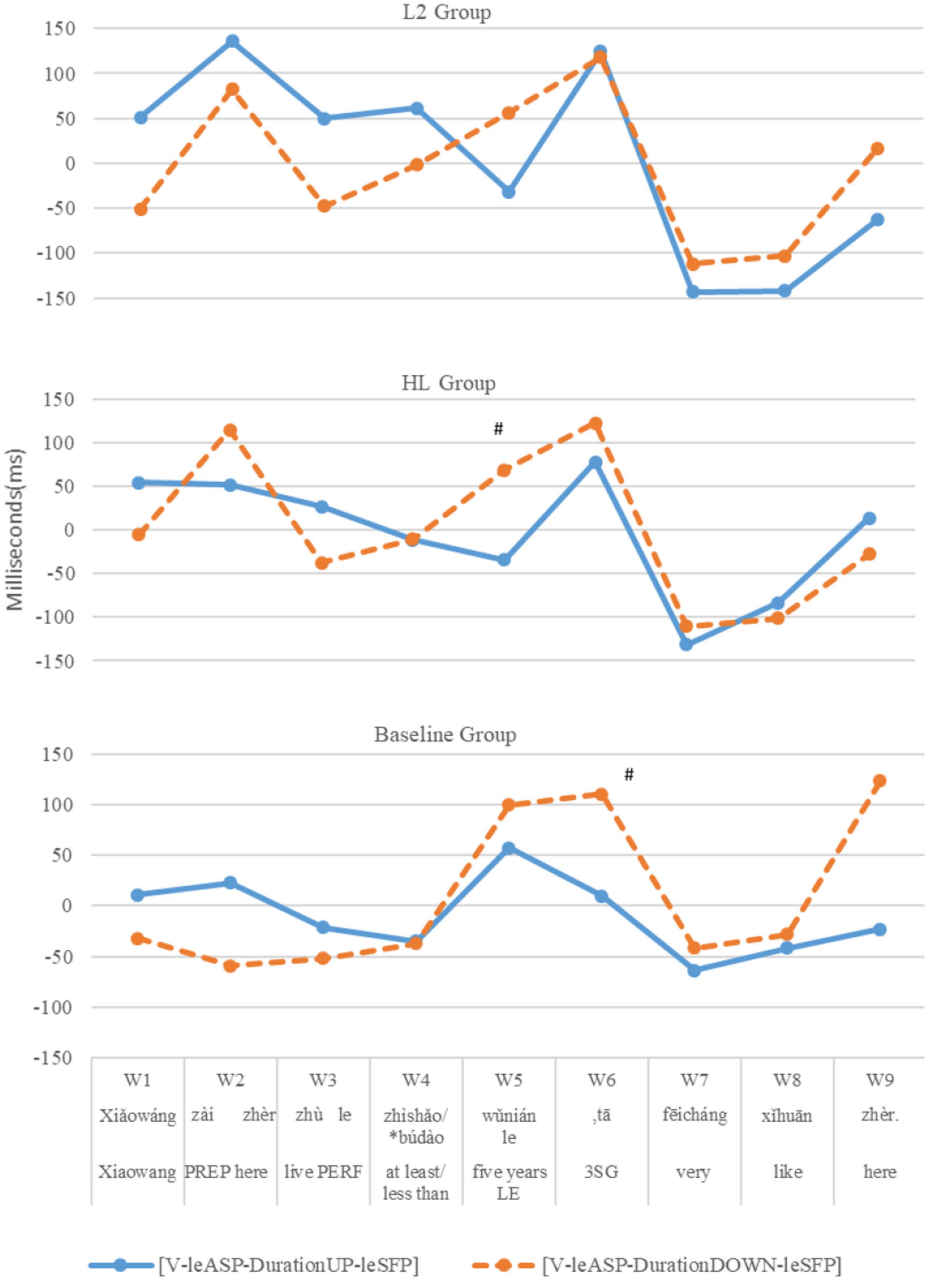
L2, HL, and baseline groups’ residual reading times at each window between [V-leASP-DurationUP-leSFP] and */?[V-leASP-DurationDOWN-leSFP].

Figure 4 presents the residual reading times at each time window in each condition by each group in three panels. Unlike the NP conditions, the three groups displayed three different patterns. As expected, baseline controls showed statistical (though borderline) differences between the upward and downward conditions in the first post-critical window (W6, *p* < 0.1, *post hoc* power = 59.72%). What is surprising is that the HLers showed statistical differences (though also borderline) earlier than the baseline controls in the critical window (W5, *p* < 0.1, *post hoc* power = 42.11%), suggesting heightened sensitivity to violations caused by the downward-entailing contexts. The L2ers showed a third pattern and did not reveal any statistical differences in reading times in any window. Table 5 presents the LMM results.

**Table 5:** Mixed-effects models (dependent variable: residual reading time at the first post-critical window (W6) for baseline, and at critical window (W5) for HLers).

	**Fixed effects**		
**Predictors**	**Estimates**	***SE***	***t***
	**HL learners**		
(Intercept)	68.76	48.01	1.43
Condition [V-*le*_ASP_-Duration_UP_-*le*_SFP_]	−102.97	61.97	−1.66#
	**Baseline**		
(Intercept)	110.67	36.98	2.99**
Condition [V-*le*_ASP_-Duration_UP_-*le*_SFP_]	−100.92	52.29	−1.93#

Reference level for condition = */?[V-*le*_ASP_-Duration_DOWN_-*le*_SFP_], Model formula for baseline group: RT ~ condition, Model formula for HL group: RT ~ condition + (1 | subject); **p < 0.01, #*p* < 0.1.

To sum up, in reading the [*be*-NP_HIGHER_-*le*_SFP_] structures, both HL and L2 groups demonstrated sensitivity to violations of the semantic constraint in (1) caused by lower NPs in online sentence reading. Their sensitivity was evidenced in the same time window (second post-critical) as that of the baseline controls. By contrast, in reading the [V-*le*_ASP_-duration-*le*_SFP_] structures, both the HLers and the baseline controls, but not the L2ers, were sensitive to violations of the semantic constraint in (1) caused by downward-entailing duration phrases. Crucially, the HLers’ sensitivity emerged very early in the critical time window during which the target SPF *le* was presented, earlier than that of the baseline controls at the first post-critical window after the target particle was presented.

## 6. Discussion

### 6.1. Summary of findings

Our research questions investigated the acquisition and processing of Mandarin SFP *le*-sentences through comparisons along the following three dimensions: (i) **structure** effect – whether learners of Mandarin are equally sensitive to violations of the semantics of the SFP *le* caused by lower-NPs in [*be*-NP-*le*_SFP_] as presented in (5) and downward-entailing elements in [V-*le*_ASP_-duration-*le*_SFP_] as demonstrated in (9); (ii) **task** effect - whether learners of Mandarin are equally successful in offline and online reading tasks; and (iii) **learner** effect – whether L2ers and HLers with matched proficiency in written Chinese are equally successful in recognizing such violations.

Our findings revealed structure, task, and the learner and their interactions all had an effect, albeit to different extents, as summarized in Table 6. In terms of **structure**, the L2ers were able to provide higher ratings for [*be*-NP-*le*_SFP_] with higher NPs than those with lower NPs in the AJ task and displayed marginally longer processing times in reading [*be*-NP-*le*_SFP_] with lower NPs than those with higher NPs; but they did not show signs of sensitivity to violations caused by the downward-entailing duration in [V-*le*_ASP_-Duration-*le*_SFP_]. The advantage of [*be*-NP-*le*_SFP_] over [V-*le*_ASP_-Duration-*le*_SFP_] in eliciting sensitivity to violations is less clear in the other two groups, who recognized violations of both structures in both tasks. With respect to **task** effects, the offline AJ task elicited either stronger or equal (but never weaker) responses to violations compared with the online SPR task. This pattern holds across groups and structures. Finally, for different types of **learners**, the HLers outperformed the proficiency-matched L2ers in that the former recognized violations in both structures and in both online and offline tasks, whereas the latter only showed target-like sensitivity to the higher/lower NPs. Most strikingly, the HLers even outperformed the baseline controls, recognizing the violation earlier during the critical time window containing the critical SFP *le*, rather than in the post-critical window.

**Table 6:** Summary of findings: sensitivity to violations of the semantics of the SFP *le.*

	[*be*-NP-*le*_SFP_]		[V-*le*_ASP_-Duration_UP_-*le*_SFP_]	
	Offline AJ	Online SPR	Offline AJ	Online SPR
L2	√√	√	X	X
HL	√√	√	√√	√%
Baseline	√√	√√	√√	√

√√ and √ = significant results at and under 0.05 and 0.10 levels respectively; X = non-significant results; % = earlier sensitivity than baseline.

### 6.2. Conditions on positive cross-linguistic influence (CLI)

Given the similarities between the SFP *le* and *already*, it is expected that the English-dominant learners of Chinese (HLers and L2ers) would benefit from this cross-linguistic similarity and should not experience difficulty in acquiring the semantic constraint attached to the SFP *le*. Our findings revealed that this positive CLI is not always present, which is consistent with previous findings in the existing literature on the absence of positive CLI in acquiring L1-L2 similar structures (Bates and MacWhinney, 1989; Papadopoulou and Clahsen, 2003; Tokowicz and MacWhinney, 2005; Jing, 2008; Sabourin and Stowe, 2008). Our findings add new evidence to the conditional nature of CLI. The structure being tested obviously played a role, as shown by our L2 learners, who demonstrated little sensitivity to the violations caused by downward-entailing duration phrases, yet performed target-like with the lower NP conditions in both online and offline tasks. Note that violations caused by the two types of structures tested in this study are subject to the same semantic constraint in (1). The L2 learners’ differential performance patterns clearly indicate that different structures under the same semantic condition in theoretical analyses could cause different problems for learners. Specifically, violations caused by the less embedded (linearly close to the target element) and semantically more transparent structures are more easily detected. This echoes the findings of Regulez and Montrul (2023) that L2 and HL acquisition are more sensitive to structural differences in the test stimuli.

So what prevents CLI from happening in this case? Despite similarities in terms of semantic meanings and conditions, the SFP *le* in Chinese and the adverb *already* in English lack apparent morphosyntactic similarity and correspondence. This adds to the difficulty for adult L2 learners to establish the necessary cross-linguistic mapping that would allow CLI to happen. In this light, positive CLI should not be taken for granted even when the L1 and L2 are similar. Furthermore, apparent morphosyntactic distance can prevent the desirable positive CLI from taking place. Pertinently, establishing the required mapping is not difficult for all Chinese-English bilinguals, which we return to in the next section.

### 6.3. Processing advantages of heritage bilinguals

The three groups of Chinese speakers in our study were all bilingual speakers of Chinese and English. On the one hand, the HLers and the baseline controls were both native and L1 speakers of Chinese; they differed in the onset age and context in which they acquired English and almost certainly in their proficiency in English, which was not tested in our study, representing a limitation. On the other, the HLers and the L2ers were both dominant in English; they also differed in the onset age and context in which they acquired Chinese. Although our cloze test, as a rapid and effective test for general proficiency in written Chinese, did not capture any difference between them, the HLers and L2ers did display considerable differences in the more challenging structure in our study (i.e., downward-entailing contexts).

We attribute the fast and accurate performance of the HLers to the advantage of co-activation of the SFP *le* and the adverb *already* in their bilingual representation. Conceivably, co-activation of Language A when processing Language B in bilinguals will either boost or compete with Language B. Since the L1-L2 structures in this study are subject to the same constraints, co-activating the English structure when processing the Chinese structure was more likely to boost and enhance, rather than damage or delay the processing. In our study, the HLers were early bilinguals who had been exposed to input in Chinese provided by native speakers of Chinese (from at least one of their parents) since birth as well as input in English provided by presumably native speakers of English in English-dominant contexts (UK, US, etc.). Among the three groups, the HLers stand out as the group that had the earliest and longest exposure to both languages and were probably the most balanced between the two languages. Crucially, English was added to the HLer’s linguistic repertoire at a time when their Chinese was still rapidly developing and highly dynamic and fluid. It is possible that cross-linguistic mapping between the SFP *le* and the adverb *already* was established during this early stage and remained stable and accessible in the early bilinguals.

The L2ers were late bilinguals with an onset age for Chinese of 18 years on average. They did not seem to reliably and consistently co-activate the similar L1 structure while reading the L2. This is consistent with the findings of Canseco-Gonzalez et al. (2010) in terms of the effects of type of bilingualism in bilingual co-activation. This further explains why the L2 learners had problems in the more complex downward-entailing contexts even in offline tasks. The baseline controls were monolingually raised in Chinese and late bilinguals of Chinese and English. Given that *already* is a high-frequency adverb in English (regardless of variety of English), they must have been exposed to this usage in the English input they had received in China. Nevertheless, they did not seem to benefit from the knowledge of the adverb *already*. Our findings provide further evidence of the learner factor in modulating positive CLI, contributing to the ongoing discussion on bilingualism co-activation. As a reviewer suggested, the individual learner’s processing strategies may also impact on their performance. We await further research to follow up on this issue.

## 7. Conclusion

This study has investigated potential positive CLI in judging and processing two grammatical structures with the SFP *le* by L2 and heritage Chinese bilinguals. Results of an offline judgment task and an online reading task show that positive CLI, though widely documented in the literature and desirable in this case, is not prevailing and is instead conditioned by the structure being tested (noun vs. verb phrase), the type of task administered (online vs. offline), and the type of the bilingual learner (early vs. late). Once in place, positive CLI and co-activation of L1 and L2 structures boost online processing proficiency, as evidenced in the superior performance of the heritage bilinguals in our study. Significantly, this study contributes to the discussion on CLI, particularly on the constraints affecting positive CLI, as well as early bilingual processing advantages.

## Data availability statement

The raw data supporting the conclusions of this article will be made available by the authors, without undue reservation.

## Ethics statement

The studies involving human participants were reviewed and approved by the Ethics Committee, Academic Committe, School of Chinese as a Second Language, Peking University. Written informed consent for participation was not required for this study in accordance with the national legislation and the institutional requirements.

## Author contributions

SY designed the study and collected the experimental data. SY and ZM analysed the data and wrote up the manuscript. YZ provided funding, advised on the design and analysis, and revised the manuscript with SY and ZM. All authors approved the submitted version.

## Funding

This work was supported by the National Social Sciences Project “Application of Chinese Proficiency Grading Standards and Strategies of Textbook Research and Development (21STA032)”.

## Conflict of interest

The authors declare that the research was conducted in the absence of any commercial or financial relationships that could be construed as a potential conflict of interest.

## Publisher’s note

All claims expressed in this article are solely those of the authors and do not necessarily represent those of their affiliated organizations, or those of the publisher, the editors and the reviewers. Any product that may be evaluated in this article, or claim that may be made by its manufacturer, is not guaranteed or endorsed by the publisher.

## Footnotes

^1^Mandarin has a perfective aspect marker *le,* which is homomorphous to the SFP *le* (Huang et al., 2009; Yan, 2023; among others). Aspect *le* follows a verb and appears before the object of the verb, if any (e.g., *Wŏ chī le píngguŏ* ‘I have eaten an apple’), whereas the SFP *le* occurs after the object at the clause-final position. Aspect *le* and the SFP *le* can co-occur and appear in [V-*le*_ASP_-O-*le*_SFP_]. In cases where the object is grammatically omitted, the distinction is less straightforward. Therefore, in our experimental stimuli to be introduced below, the SFP *le* always follows an overt object of the verb [e.g., *Wŏ chī píngguŏ le* ‘I have eaten apples, (which is not the case before)’].

^2^As suggested by a reviewer, we consulted the Center for Chinese Linguistics corpus at Peking University to evaluate the semantic restrictions in (1) in native naturalistic speech samples. We used the upward/downward-entailing elements (*zhìshăo*, *búdào*) and higher/lower NPs (*dà-NP*, *xiăo-NP*) as target words and identified 99 tokens *zhìshăo* + SFP *le* (out of 2,487 tokens including the target words) and 114 tokens of *dà* ~ + SFP *le* (out of 1,135 tokens including the target words). By contrast, no downward-entailing **búdào* + SFP *le* was found. We also found 49 cases of lower NPs *xiăo* ~ + SFP *le*. Nevertheless, the lower NPs were all within the scope of negators (e.g., *búshì xiăo háizi*, ‘NEG-be a small child’), which identifies a higher point on the scale and is thus consistent with the generalization in (1).

^3^Our own informants, who were monolingual speakers of Min from Zhangzhou, Xiamen, and Taiwan, identified *liau* in Min Chinese as the equivalent of the Mandarin SFP *le*.
