# Supplementary material for: Positive cross-linguistic influence in the representation and processing of sentence-final particle le by L2 and heritage learners of Chinese
Source: Front Psychol. 2023 Jul 25;14:1145493. doi: 10.3389/fpsyg.2023.1145493 (PMC10408456; doi:10.3389/fpsyg.2023.1145493)
Supplement: Supplementary file 1 [file Data_Sheet_1.PDF]

ID	Languages exposure				
	Language(s)	From whom	How often		
			0-3 year	4-6 year	7-12 year
1	Mandarin	parents	daily	daily	daily
2	Mandarin	mother	seldom	often	often
		relatives+family friends	occasionally	occasionally	occasionally
3	Cantonese	parents	always	always	always
4	Mandarin	parents	always	always	often
5	Min	parents	always	always	always
6	Mandarin	parents	half time	half time	half time
		grandparents	always	always	always
7	Mandarin	parents	often	often	often
		grandparents	always	always	always
8	Mandarin	parents	mom: always; dad: sometimes	mom: always; dad: sometimes	mom: always; dad: sometimes
9	Mandarin, Cantonese, Min	parents	always	always	always
10	Cantonese	parents	mom: always; dad: rarely	mom: always; dad: sometimes	mom: always; dad: rarely
11	Mandarin	parents	mostly	mostly	mostly
12	Mandarin	parents	always	always	always
13	Mandarin	mother	often	often	often
14	Mandarin	mother	always	always	always
15	Mandarin	parents	very often	very often	very often
16	Mandarin	parents	always	mom: always; dad: seldom	always
17	Mandarin	mother+grandparents +relatives	always	always	always
18	Mandarin	parents	always	always	always

19	Mandarin	parents	most of the time	most of the time	mom: half time, dad: half time
